# Is dual cup mobility better than hemiarthroplasty in patients with dementia and femoral neck fracture? A randomized controlled trial

**DOI:** 10.1051/sicotj/2019035

**Published:** 2019-11-01

**Authors:** Raffaele Iorio, Ferdinando Iannotti, Daniele Mazza, Attilio Speranza, Carlo Massafra, Matteo Guzzini, Carmelo D’Arrigo, Andrea Ferretti

**Affiliations:** Sapienza University, Sant’Andrea Hospital Rome 00189 Italy

**Keywords:** Dual mobility cup, Dementia, Femoral neck fracture, Hemiarthroplasty, Dislocation

## Abstract

*Purpose*: Treatment of patients with dementia and hip fracture is challenging. Total hip arthroplasty (THA) with dual mobility cup (DMC) has been designed to reduce the rate of dislocation by increasing the stability of the implant. This study aimed to compare the dislocation rates of DMC THA with hemiarthroplasty (HA) in elderly patients with displaced femoral neck fracture (FNF) and a diagnosis of dementia.

*Methods*: All patients with a displaced FNF and dementia diagnosis were prospectively randomized to hemiarthroplasty or THA with DMC treatment during a 2-year period. Finally, the outcomes of 30 patients in the HA group were compared with those of 30 patients in the DMC THA group. Dislocation rate at a minimum follow-up of 1 year was evaluated as the primary outcome. Reoperation rate, time to surgery, surgical time, length of hospital stay, and 30-day and 1-year mortality were also evaluated.

*Results*: There was a significant difference regarding rates of dislocation in favor of THA with DMC and with regard to length of surgery (*p* = 0.04) in favor of bipolar HA. Dislocation occurred in five patients (16.6%) treated with bipolar HA and no one (0%) in patients treated with THA with DMC (*p* = 0.019). There was no difference with regard to the 30-day mortality, 1-year mortality, reoperations, and length of hospital stay between the two groups of patients.

*Discussion*: THA with DMC seems to be a safe and reliable choice to reduce the rate of dislocation at 1 year in patients with dementia and FNF without a higher risk of mortality.

## Introduction

The elderly population is increasing, and consequently, the incidence of hip fractures is rising [1]. The worldwide annual number is predicted to be up to 6.26 million by the year 2050 [2].

Elderly patients are fragile and may have several comorbidities [3]. Dementia is a clinical syndrome characterized by cognitive disability [4], balance problems, impaired mobility, and a consequent increased risk of falls. Moreover, these patients are more likely to have osteoporosis; accordingly, a significant association has been detected between dementia and the increased risk of femoral neck fracture (FNF) [5].

A higher mortality and morbidity rates were found in patients with FNF; moreover, a correlation between fragility fractures and loss of self-sufficiency was identified, it is due to a worsening of motor skills and function [2].

Treatment of patients with dementia and FNF is demanding; these kinds of patients are unable to follow postoperative motion restrictions, and they may have a higher risk of hip dislocation [6].

Hip instability accounts for 22.5% of surgical revisions being the most common cause of THA revision not only in the United States [7] but also all over the world.

Which is the best treatment for displaced FNF is still debated [8]. Bipolar Hemiarthroplasty (HA) is associated with lower dislocation rate, shorter surgical time, and lower blood loss than THA; on the other hand, THA has better functional outcomes, lower reoperation rate, and lower long-term costs [9–12].

Dual mobility cup (DMC) THA was introduced in the 1970s by Gilles Bousquet. He combined the reduced wear of the small femoral head with the greater stability guaranteed by a large mobile liner that increases the effective diameter of the head [13, 14]. DMC was used as an alternative to bipolar arthroplasties to avoid also acetabular protrusion. Current designs are developed to reduce the risk of dislocation and increase the range of motion. [15].

This study aimed to compare the dislocation rates of DMC THA with hemiarthroplasty (HA) in elderly patients with displaced FNF and a diagnosis of dementia. Time to surgery, surgical time, reoperation rate, 30-day and 1-year mortality, and length of hospital stay were also evaluated.

## Materials and methods

All patients with a displaced intracapsular fracture of the hip and dementia diagnosis were eligible for inclusion during a 2-year period beginning October 2015. Inclusion criteria applied were: displaced FNF (Garden 3 or 4), dementia diagnosis made by a professional Geriatric Assessment Team, to the DSM-5 criteria, and with a Mini-Mental Test score < 18, patients aged more than 60 years able to walk unaided before fracture. Exclusion criteria were: pathological fracture secondary to malignant disease and concomitant fracture requiring surgery.

Patients meeting the eligibility criteria were enrolled in the study after informed consent was obtained. Patients were prospectively randomized to HA or THA with DMC treatment with an alternate assignment on the basis of their order of admission. In the hemiarthroplasty group, patients receive an Excia cementless femoral stem with bipolar head (B. Braun, Aesculap, Tuttlingen, Germany); in the THA group, patients receive a dual mobility cup Quattro (Groupe Lépine, Genay, France) with Pavi cementless femoral stem (Groupe Lépine, Genay, France). All patients received antibiotic and venous thromboembolic prophylaxis in accordance with local protocols. A direct lateral approach was performed in all patients in lateral decubitus. On the second day, weight-bearing was allowed, and a guided rehabilitation protocol with standard motion restrictions was prescribed.

Preoperative data recorded were: age at surgery, gender, time to surgery, and American Society of Anaesthesiology (ASA) score. Intraoperative data consisted of operating time. The dislocation rate at a minimum follow-up of 1 year was evaluated as the primary outcome. Reoperation rate, time to surgery, surgical time, length of hospital stay, and 30-day and 1-year mortality were regarded as secondary outcomes.

### Statistical analysis

Statistical analysis was performed using Intercooled Stata 9.0 (Stata Inc., College Station, TX, USA). Student’s *t*-test was used to analyze the differences between continuous data, while the chi-square test was used for categorical data. Statistical significance was defined for values of *p* < 0.05.

## Results

**Figure 1:**
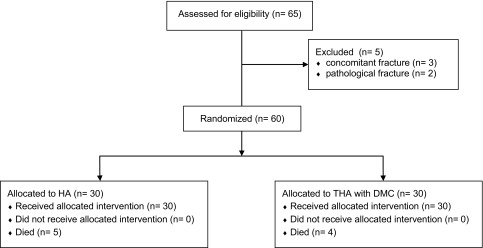
Participant flowchart of the enrollment.

After screening 65 patients, 5 patients were excluded and 60 were enrolled in the study; 30 were randomized to the HA group and 30 to the THA with DMC group. Nine patients died of causes unrelated to the procedure within the first year (five in the HA group, four in the DMC THA group). A summary of the flow of patients through the study is outlined in Figure 1.

No statistically significant differences were found regarding demographic data between groups (Table 1).

**Table 1:** Demographics data.

	Hemiarthroplasty	THA with DMC	*P* value
Gender (*n*)			
Male	13	12	>0.05
Female	17	18	>0.05
Mean age (*SD*)	83 (3)	82 (4)	>0.05
ASA score (*n*)			
2	4	3	>0.05
3	21	23	>0.05
4	5	4	>0.05
Median time to surgery, h (min.−max.)	51 (12–72)	59 (16–68)	>0.05

Primary and secondary outcomes are outlined in Table 2. There was a statistically significant difference with regard to the dislocation rate in favor of DMC THA; five dislocations (16.6%) were detected in the HA group and none (0%) in the DMC THA group (*p* = 0.019). Dislocations with bipolar HA happened within the first 60 days after surgery. One patient in the HA group underwent reoperation due to a local infection while no reoperation (0%) occurred in the THA with DMC group.

**Table 2:** Primary and secondary outcomes.

	Hemiarthroplasty	THA with DMC	*P* value
Dislocation (%)	5 (16.6%)	0 (0%)	0.019
Re-operation	1	0	>0.05
Length of surgery, min (min.–max.)	48 (42–63)	59 (45–74)	0.04
Duration of stay, days (min.–max.)	5.5 (5–7)	6.1 (5–8)	>0.05
30 days mortality (%)	1 (3.3%)	1 (3.3%)	>0.05
1 year mortality (%)	5 (16.6%)	4 (13.3%)	>0.05

Length of surgery was significantly shorter for HA (48 min) than for THA with DMC (59 min) (*p* = 0.04).

There was no difference regarding 30-day mortality, 1-year mortality, and length of hospital stay between groups.

## Discussion

Results from this study highlight that THA with DMC may be a better solution than HA in patients with dementia; we reported promising results with regard to dislocation and reoperation rate after DMC THA.

To our knowledge, this is the first RCT comparing DMC with hemiarthroplasty in a selected population of patients with dementia and FNF.

Patients with dementia are about 35 million worldwide, and they will tend to double in the next 20 years [5]. Hip fracture surgery is one of the most performed surgeries in demented patients [16] with a significantly higher risk of postoperative complications especially in the elderly [4].

Due to mental impairment, hip instability after surgery may be a frequent condition in these patients; however, this information is lacking [6].

Very few papers analyzed the results of DMC in patients with a high risk of dislocation [8, 17, 18] and only one study in patients with dementia [6].

Caton et al. reported a low rate of dislocation in primary cemented Charnley-type THA with DMC (0.9%) compared with standard polyethylene cup (12.9%) concluding that this surgical choice seems to be safe and effective especially in a high-risk population [19].

Sanders et al. [20] reported no dislocations using the AVANTAGE^®^ DMC in eight patients (10 hips) with cerebral palsy at a mean follow-up of 39 months.

Graversen et al. [6] showed no case of dislocation and revision after primary hip arthroplasty with DMC in patients with dementia and FNF at 1-year follow-up.

Our results are similar; moreover, we found a significantly higher rate of dislocation in patients treated with HA.

HA is a widely used treatment with low dislocation rates (3–3.4%) in elderly non-selected patients with FNF [8, 21]; however, functional outcomes after HA seem to be worse than THA, as well as pain [12].

Acetabular erosion and reoperation rate may be higher for HA than for THA; there were no differences regarding infection rate, complications, and first-year mortality [11].

Assi et al. reported a very low first-year mortality rate of 1.7% after THA with DMC in patients with FNF; they recorded no dislocations, infections, or aseptic loosening at a mean follow-up of 61.2 months [22].

The two articulations in the DMC have raised concerns regarding the risk of polyethylene wear and aseptic loosening; however, recent studies showed equivalent wear between DMC and standard cups [23]. Anyhow, in elderly patients with dementia and low activity level, a painless THA with a lower dislocation and reoperation risk seems more important than the risk of polyethylene wear [6].

Noyer and Caton indicate that wear involves also the third articulation between stem neck and insert rim [24]. Prudhon et al. showing contemporary DMC results, emphasize the importance of Charnley stem to reduce the risk of impingement and wear on the retentive rim and to prevent intra-prosthetic dislocation. They suggest that DMC has improved outcomes and could be considered as “the new gold standard in THA” [25].

Limitations of this study are the lack of functional outcomes as well as patients reported outcome measures (PROMs), then the small groups, but it’s due to the main inclusion criteria: dementia.

Total hip arthroplasty (THA) with DMC seems to be a safe and reliable choice to reduce the rate of dislocation at 1 year in patients with dementia and displaced FNF without a higher risk of mortality.

## Conflicts of interest

The authors declare that they have no competing interest.
